# RelA Is an Essential Target for Enhancing Cellular Responses to the DNA Repair/Ref-1 Redox Signaling Protein and Restoring Perturbated Cellular Redox Homeostasis in Mouse PDAC Cells

**DOI:** 10.3389/fonc.2022.826617

**Published:** 2022-03-24

**Authors:** Mahmut Mijit, Randall Wireman, Lee Armstrong, Silpa Gampala, Zonera Hassan, Christian Schneeweis, Guenter Schneider, Chi Zhang, Melissa L. Fishel, Mark R. Kelley

**Affiliations:** ^1^ Department of Pediatrics, Herman B Wells Center for Pediatric Research, Indiana University School of Medicine, Indianapolis, IN, United States; ^2^ Department of Clinic and Polyclinic for Internal Medicine II, Klinikum rechts der Isar, Technical University of Munich, Munich, Germany; ^3^ Department of General, Visceral and Pediatric Surgery, University Medical Center Göttingen, Göttingen, Germany; ^4^ Department of Medical and Molecular Genetics, Indiana University School of Medicine, Indianapolis, IN, United States; ^5^ Department of Biohealth Informatics, Indiana University-Purdue University (IUPUI), Indianapolis, IN, United States; ^6^ Indiana University Melvin and Bren Simon Comprehensive Cancer Center, Indiana University School of Medicine, Indianapolis, IN, United States; ^7^ Department of Pharmacology and Toxicology, Indiana University School of Medicine, Indianapolis, IN, United States; ^8^ Department of Biochemistry and Molecular Biology, Indiana University School of Medicine, Indianapolis, IN, United States; ^9^ Indiana University Melvin and Bren Simon Comprehensive Cancer Center, Indiana University, Indianapolis, IN, United States

**Keywords:** pancreatic ductal adenocarcinoma (PDAC), Ape1, transcriptional factors, relA, DNA repair, redox signaling, PRDX1, STAT3

## Abstract

Pancreatic ductal adenocarcinoma (PDAC) is one of the deadliest cancers with a poor response to current treatment regimens. The multifunctional DNA repair-redox signaling protein Ref-1 has a redox signaling function that activates several transcriptional factors (TFs) including NF-κB (RelA), STAT3, AP-1. These have been implicated in signaling in PDAC and associated with cancer progression and therapy resistance. Numerous studies have shown a role for RelA in PDAC inflammatory responses and therapy resistance, little is known as to how these inflammatory responses are modulated through Ref-1 redox signaling pathways during pancreatic pathogenesis. RelA and STAT3 are two major targets of Ref-1 and are important in PDAC pathogenesis. To decipher the mechanistic role of RelA in response to Ref-1 inhibition, we used PDAC cells (KC3590) from a genetically engineered *Kras* ^G12D^-driven mouse model that also is functionally deficient for RelA (Parent/Vector) or KC3590 cells with fully functional RelA added back (clone 13; C13). We demonstrated that RelA deficient cells are more resistant to Ref-1 redox inhibitors APX3330, APX2009, and APX2014, and their sensitivity is restored in the RelA proficient cells. Knockdown of STAT3 did not change cellular sensitivity to Ref-1 redox inhibitors in either cell type. Gene expression analysis demonstrated that Ref-1 inhibitors significantly decreased IL-8, FOSB, and c-Jun when functional RelA is present. We also demonstrated that PRDX1, a known Ref-1 redox modulator, contributes to Ref-1 inhibitor cellular response. Knockdown of PRDX1 when functional RelA is present resulted in dramatically increased PDAC killing in response to Ref-1 inhibitors. The enhanced cell killing was not due to increased intracellular ROS production. Although Ref-1 inhibition decreased the NADP/NADPH ratio in the cells, the addition of PRDX1 knockdown did not further this redox imbalance. This data suggests that the mechanism of cell killing following Ref-1 inhibition is at least partially mediated through RelA and not STAT3. Further imbalancing of the redox signaling through disruption of the PRDX1-Ref-1 interaction may have therapeutic implications. Our data further support a pivotal role of RelA in mediating Ref-1 redox signaling in PDAC cells with the *Kras* ^G12D^ genotype and provide novel therapeutic strategies to combat PDAC drug resistance.

## Introduction

Pancreatic ductal adenocarcinoma (PDAC) is one of the deadliest cancers due to poor response to current treatment regimens and lack of markers for early diagnostics, resulting in a 5-year overall survival of around 10% (1). Kras mutation is the most dominant oncogenic transformation in PDAC mutational profile confirmed in ∼90% of cases (2). The oncogenic Kras mutation leads to alteration of signaling pathways that are associated with the progression and metastasis of PDAC and is the main contributor of therapy recalcitrance (3).

Inflammation and remodeling of the local tumor microenvironment (TME) are key cellular events that exacerbate progression of PDAC. Aberrant Kras signaling activates several inflammatory signaling pathways, e.g., NF-κB, AP-1, IL-6/STAT3 signaling, that are constitutively active in PDAC and highly expressed in PDAC and its TME (4). Kras^G12D^ induces IL-1α expression *via* AP-1 activation, leading to NF-κB activation in tumor cells (5). Elevated levels of cytokines and chemokines are also observed in PDAC and correlated with the enhanced NF-κB signaling (6). Inhibition of NF-κB signaling in cancer-associated fibroblasts (CAFs), a major constituent of the TME, abolished its tumor-promoting effects, suggesting that NF-κB is critically involved in PDAC and the TME (7). Increasing evidence demonstrates that activated NF-κB partners with other signaling molecules, such as STAT3 and HIF-1α, and induces chemoresistance to gemcitabine and platinum agents, first line therapeutic regimens for PDAC (8–10). Consequently, the main challenge from the perspective of cancer treatment is identifying key molecular players that mediate cellular responses and are effective on PDAC cells with the activated Kras genotype.

Apurinic/apyrimidinic endonuclease-1/redox factor-1 (APE1/Ref-1 or Ref-1) is a multifunctional protein active in DNA repair, redox-signaling (reduction/oxidation) control, and transcriptional regulatory activities (11). Ref-1 functions in DNA base excision repair (BER) by virtue of its endonuclease activity and responds to oxidative and alkylation DNA damage lesions. Ref-1 is also involved in redox signaling through a thiol exchange reaction (12, 13). The Ref-1 redox activity reduces critical cysteine residues on transcription factors (TFs), such as RelA (subunit of NF-κB), AP-1, HIF-1α, STAT3 leading to transcription factor activation. Activation TFs that are regulated by Ref-1 have been implicated in tumor growth and proliferation, metastasis, metabolism, and survival of tumor cells as well as signaling within the TME (14). Ref-1 redox activity can be regulated by direct interactions with other proteins such as Peroxiredoxin 1 (PRDX1) or thioredoxin 1 (TRX1) and constitute the PRDX1/Ref-1/TRX1 redox regulatory cycle in cells (15, 16). For example, the redox interactions between NF-κB, PRDX1 and Ref-1 are responsible for overproduction of inflammatory cytokine, IL-8 (15). Several *in vitro* studies demonstrated significant inhibition of DNA binding activity of RelA and its altered subcellular localization when cells were challenged with small molecules (APX3330, APX2009, and APX2014) that target the redox signaling function of Ref-1 (17–19). The latter two are more potent second-generation Ref-1 redox inhibitors (20). Blocking the redox activity of Ref-1 using APX3330 results in inhibition of TNF-α-induced activation of IL-8 production in human cancer cell lines (17). However, the relationship of PRDX1-Ref-1 and subsequent RelA activation has not been rigorously explored beyond initial studies.

Previous work reported that RelA possesses dual functional roles during pancreatic oncogenesis, by promoting tumor suppression through regulation of inflammatory cytokines or facilitating proliferation of transformed tumor cells and tumor progression through bypassing senescence (21). However, the mechanistic details of how redox signaling regulates RelA-driven cellular proinflammatory events that drive therapy resistance or exploiting these events in cancer treatment remain to be investigated. In the present study, we focused on the cellular inflammatory responses of Ref-1 redox signaling inhibition in a murine PDAC *in vitro* model. Specifically, we wanted to investigate the relationship of RelA-dependent cellular responses to Ref-1 redox signaling inhibition and further identify other possible associated molecular targets or signaling pathways that may enhance cellular sensitivity to Ref-1 redox signaling inhibition. The model used is a mouse PDAC cell line generated from a Kras^G12D^-driven mouse model that also is functionally deficient for RelA (KC3590: Parent/Vector) (22). A fully functional RelA was added back to these KC3590 cells creating two clones (C13/C15) (23). We used these KC3590 cell lines to examine the roles of known Ref-1 target, RelA and known interacting protein of Ref-1, peroxidredoxin 1 PRDX1 and their effects on cellular sensitivity, ROS, and redox state *via* NADPH/NADP ratio to Ref-1 inhibitors. Our data demonstrate an essential involvement of Ref-1 redox signaling in RelA-driven cellular responses in PDAC cells with the Kras^G12D^ genotype such that targeting Ref-1 may be a promising strategy to improve acquired resistance in PDAC chemotherapy.

## Material And Method

### Cell Culture and Cell Lines

We used KC3590, a mouse PDAC cell line that was established from *Ptf1a^Cre/+^;LSL-Kras^G12D/+^;p65^loxP/loxP^* mice, with RelA truncation at exon (7-10). This truncation only codes for part of the Rel homology domain and the nuclear localization site and is inactive (22). Thus, KC3590 Parent and Vector lines express non-functional RelA (hereafter referred to as Parent or Vector). KC3590 cells were transfected with pcDNA3-Flag-RelA (prepared by Dr. Smale) (23) and are referred to as Clone #13 (C13) and Clone #15 (C15). PDAC mouse cell lines referred to as KC6075, KC8442, KC2259, KC53631, KC9091, KC5671, KC5559, KC5748 were isolated from tumors in mice that carry a Pdx1-Cre recombinase oncogenic *Kras ^G12D^* mutation. All cell lines were maintained at 37°C in 5% CO_2_ and grown in Dulbecco’s Modified Eagle’s Medium (DMEM, Invitrogen, Carlsbad, CA, USA) with 10% Fetal Bovine Serum (FBS) (Atlanta Biologicals, Minneapolis, MN, USA). The cell lines were authenticated by STTR analysis and tested negative for mycoplasma contamination.

### Ref-1 Inhibitors

Small molecule inhibitors were prepared and used as previously described (24). Ref-1 redox signaling was inhibited using APX3330, APX2009, and APX2014 (Apexian Pharmaceuticals; Indianapolis, IN). RN7-58 (Apexian Pharmaceuticals) was used as a negative control and is structurally similar but does not inhibit Ref-1 redox signaling activity (25). APE1 repair inhibitor III (ARi3) (EMD Chemicals, CA, USA) was used as an inhibitor of the endonuclease activity of Ref-1 (26, 27).

### Cell Viability and APE1 Redox Inhibitors Cytotoxicity

Cell proliferation and viability were measured with alamarBlue Cell Viability assay (Invitrogen, Eugene, USA) as previously described (25). Briefly, cancer cell lines were seeded at 2000 cells/well in 96-well tissue culture plates and their growth rates monitored. Cell viability was measured 48 hours after treatment and response was normalized to a non-treated (media only or vehicle) control. At least three replicates were performed.

### siRNA Transfections

Targeted mRNA knock-down was optimized for each cell line and verified by Western blot (>80% knockdown) as previously reported (28). Cells were transfected by lipofectamine RNAiMax (Invitrogen, CA, USA) with PRDX1 (SR405074, OriGene Technologies, MD, USA), RelA (SR417160, OriGene Technologies, MD, USA), STAT3 (SR427487, OriGene Technologies, MD, USA), and universal scrambled control (SCR) (SR30004, OriGene Technologies, MD, USA) siRNAs.

After 24 hours post-transfection, cells were re-plated into 96-well plates for assessing Ref-1 inhibitors cytotoxicity.

### RNA Isolation, Reverse Transcription, and Real-Time Quantitative PCR (qRT-PCR)

Cells were collected and processed for RNA extraction according to the manufacturer’s protocol (Qiagen, Hilden, Germany, USA). The RNA concentrations were determined using a NanoDrop (Thermo Fisher, MA, USA). Subsequently, 1μg of RNA/25-μl reaction mix was reverse-transcribed to cDNA using random hexamers and MultiScribe reverse transcriptase (Applied Biosystems, Foster City, CA). qRT-PCR was performed in 96-well plates, with a final volume of 20 μL/well using the SYBR Green PCR kit (Applied Biosystems, Foster City, CA, USA) on the CFX96 real-time PCR detection system (BioRad, Hercules, CA, USA). Primers for indicated genes are commercially available (OriGene, Technologies, MD, USA) and primers sequence are shown in supplemental data (**Supplemental Data: Table S1**). qRT-PCR cycling conditions were 1 min at 95°C, 10 min at 95°C, 15 s at 95°C and 1 min at 60 °C for 40 cycles. Relative changes in mRNA expression levels were assessed by the 2^-ΔΔCT^ method, and changes in mRNA expression of the target gene were normalized to that of RPL6 gene, as previously published (25, 29).

### Total Protein Extraction and Western Blotting Analysis

Whole extracts from control and treated cells were obtained in 1% SDS extraction buffer supplemented with protease inhibitors (Santa Cruz Biotechnology, TX, USA). Briefly, cell extract was heated at 95°C for 5min, then sonicated (4 pulses, 4 cycle) to shear the DNA in the samples. Total protein concentration was determined by using the DC Protein Assay Kit (Bio-Rad Laboratories, CA, USA), and bovine serum albumin (BSA) (Bio-Techne, MN, USA) as the standard. Denatured samples (25–40 μg) were subjected to SDS-PAGE and proteins were transferred onto nitrocellulose membranes by electrophoretic transfer. Non-specific binding sites were blocked at room temperature for 1h with 5% (w/v) Blotting-Grade Blocker (Bio-Rad Laboratories, CA, USA) in Tris-buffer saline (Boston BioProducts, MA, USA) containing 0.05% (v/v) Tween-20 (Thermo Fisher, MA, USA) (TBS-T). Membranes were incubated overnight with the primary antibodies, anti-PRDX1(66820-1, Protientech), anti-STAT3 (CS-9139, Cell signaling), anti-Ref-1 (13B8E5C2, Novus Biologicals), anti-RelA (sc-8008, Santa Cruz) and anti-Vinculin (CP74-100, Millipore) diluted in TBS-T (1:1000), and then, with the peroxidase-conjugated secondary antibody (1706516, Bio-Rad Laboratories, CA, USA) for 1 h. Signal was then captured by using Bio-Rad ChemiDoc imager, and band intensities were analyzed by densitometry on Image Lab software (Bio-Rad Laboratories, CA, USA). Vinculin expression was used as loading controls and used for data normalization.

### Intracellular ROS Assays

KC3590 cells were transfected with target siRNA (PRDX1) as described above. Cells were collected at 24 hours post transfection and seeded at 10,000-12,000 cells/well in 96-well plates. At 48 hours post transfection (80-90% confluency), cells were treated with Ref-1 redox inhibitors, APX3330, APX2009, APX2014, as well as vehicle (DMSO) and media controls; all constituted in Opti-MEM (ThermoFisher, MA, USA) and treated for 2 hours at 37°C, 5% CO_2_. CellROX® Green Reagent (Molecular Probes, Oregon, USA) was added to the drug media to a final concentration of 5 μM and incubated with reagent for 30 minutes. Next, media was removed, and one PBS wash was performed. ROS fluorescence was detected at 485/528nm excitation/emission (BioTek Synergy H4).

### NADP/NADPH Assay

NADPH to NADP ratio in KC3590 cells was measured using NADP/NADPH Assay Kit from Abcam (Abcam, Inc, Cambridge, UK). Assay was performed as per manufacturer’s protocol. Briefly, after treatment with Vehicle control or APX2009, 4 × 10^6^ cells were lysed using 400 μL extraction buffer for cytoplasmic NADPH/NADP. Samples were sheared and passed through DNA spin columns. 150 μL of extracted samples were heated to 60 °C for 30 min to decompose NADP leaving NADPH and the remaining sample was used for total NADP (NADPt). 50 μL of standard or sample was used per well. 100 μL of Reaction Mix was added and incubated for 5 min at room temperature followed by addition of 10 μL of NADPH Developer per well. The readings were taken at OD_450nm_. NADPH/NADP ^+^ was calculated as NADPH/NADP + ratio = NADPH/(NADPt—NADPH). The measured NADP and NADPH levels were calculated by comparison with a standard curve.

### Statistics

All the experiments were performed at least three independent times. The obtained data were expressed as ‘Mean + Standard Error’. Significance was calculated as per either One-way ANOVA or two-way ANOVA multiple comparisons wherever applicable using Graph Pad Prism Version 9. The difference was considered statistically significant when p-value < 0.05. For qRT-PCR, analysis of covariance models (ANCOVA) was performed to test the Ct difference of each target gene value between treatment with APX3330, APX2009 and vehicle (DMSO) after standardization by reference gene (RPL6) (29). A p-value of at least < 0.05 was considered statistically significant.

## Results

### Re-Expression of RelA Renders Tumor Cells Sensitive to Ref-1 Inhibitors

Initially, we investigated how various murine PDAC tumor cells driven by Kras^G12D^ mutation would respond to Ref-1 inhibition. A panel of cell lines established from the *Kras^G12D^*; Pdx1-Cre (KC) mice were screened for sensitivity to APX3330, APX2009, and APX2014 and ranked based on inhibitor sensitivity (**Supplemental Data: Table S2**). We demonstrated that there are significant differences (p<0.001) in cell viability between the most resistant cell line (KC6075) and the most sensitive cell line (KC5748) when cells were challenged with indicated concentrations of Ref-1 inhibitors, APX3330 (25, 50, 75μM), APX2009 (3.1, 6.25, 12.5 μM) and APX2014 (6.25, 12.5, 25μM) (**Supplemental Data: Figure S1**).

**Figure 1:**
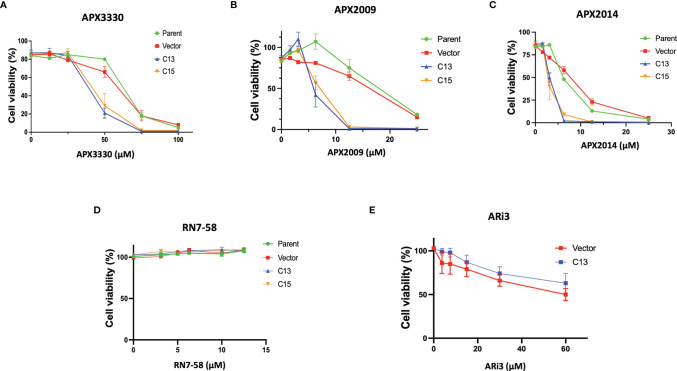
Re-expression of RelA renders tumor cells sensitive to Ref-1 inhibitors. KC3590 cells that underwent functional deletion of RelA (Parent and Vector) and KC3590 clone lines that express functional RelA (C13, C15) were challenged with different concentrations of APX3330 **(A)**, APX2009 **(B)** and APX2014 **(C)**, RN7-58 **(D)**, ARi3 **(E)** for 48 hours. Cytotoxicity was measured by Alamar blue (4 hr incubation) at 590 nm. Error bars represent standard error (N = 3). Statistical differences between EC_50_s of Ref-1 inhibitors in KC3590 cells are provided in **Table 1**.

To investigate Ref-1’s regulation of RelA in PDAC cells, matched RelA deficient and proficient KC lines were utilized. Blockade of NF-κB/RelA signaling is important due to its role in driving differential sensitivity to chemotherapy agents, such as gemcitabine during PDAC oncogenesis. To examine the mechanistic role of RelA in response to Ref-1 inhibition, KC3590 cell lines were treated with APX3330, APX2009, APX2014. We found that KC3590 RelA-deficient cells (Parent, Vector) were 1.7-2.5–fold more resistant to Ref-1 inhibitors than RelA-proficient cells (C13, C15) (**Figures 1A**–**C**). EC_50_s of Ref-1 inhibitors in KC3590 cells are shown in **Table 1**. In contrast, we did not observe any significant cellular sensitivity when KC3590 cells were treated with Ref-1 inactive redox inhibitor analog, RN7-58 (**Figure 1D**) or with ARi3, an inhibitor of Ref-1 endonuclease activity (**Figure 1E**). Overall, RelA deficient cells demonstrated two-fold more resistance to Ref-1 inhibitors, and their sensitivities were restored in cells expressing functional RelA. This suggests that at least some of the cell killing following Ref-1 inhibition is mediated through RelA and on-target effects of the APX compounds as the effects of the inhibitors are more dramatic in cells that are RelA proficient.

**Table 1:** EC_50_ (µM) summary table of APX3330, APX2009, APX2014 cytotoxicity assays in KC3590 lines.

EC_50_ µM	Parent	Vector	C13	C15
**APX3330**	60.8 (± 1.5)	52.9 (± 5.3)	^*^36.2 (± 2.3)	^*^39.3 (± 5.7)
**APX2009**	16.8 (± 1.9)	13.7 (± 1.1)	^***##^6.6 (± 0.1)	^**##^6.6 (± 0.2)
**APX2014**	6.0 (±0.3)	6.0 (±0.9)	^***###^3.0 (± 0.1)	^***###^2.8 (± 0.4)

*p < 0.05, **p < 0.001, ***p < 0.0001 vs Parent line; ^##^p < 0.001, ^###^p < 0.0001 vs Vector line, One-way ANOVA (N = 3).

### Ref-1 Inhibitors Suppress Inflammatory Responses *via* RelA Mediated Pathways

As an indicator of RelA activity, we assessed the levels of three genes (IL-8, FOSB, and c-Jun) in the RelA deficient and proficient KC lines after treatment with Ref-1 inhibitors (30). IL-8 is a well-established RelA target gene while FOSB and c-Jun are components of the AP-1 family of proteins, a transcriptional target of Ref-1. The AP-1 and RelA TFs have also been shown to crosstalk and influence expression of various AP-1 family members (30). Single cell RNA sequencing data from human PDAC cells revealed that FOSB and c-Jun were strongly downregulated with Ref-1 knockdown (**Supplementary Figure S3**). To investigate the interplay with Ref-1, AP-1, and RelA and these genes in response to Ref-1 inhibitors, we assessed Il-8, FOSB, and c-Jun mRNA levels both in RelA-proficient and deficient cells following Ref-1 inhibitor treatment.

**Figure 2:**
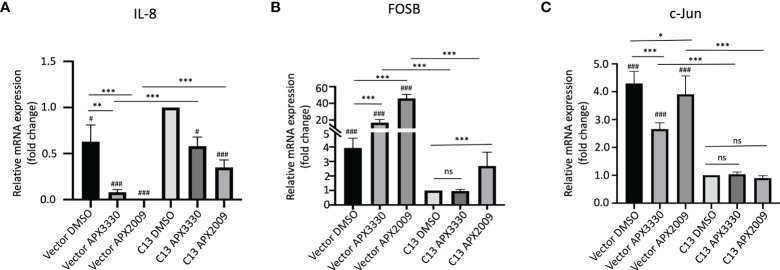
Ref-1 inhibitors suppress inflammatory responses *via* RelA mediated pathways. KC3590 cells that are RelA deficient (Vector) and clone with functional RelA add back (C13) were challenged with Ref-1 inhibitors, APX3330 (60 μM Vector; 30 μM C13), APX2009 (16 μM Vector; 6 μM C13) based on their EC50 for 24 hours. The effects of Ref-1 inhibitors on mRNA expression levels of IL-8, FOSB, c-Jun was assessed with qPCR (Figure **(A–C)**, respectively). Six independent experiments were performed (n = 6). Hashtag “#” is comparing to C13 DMSO, ^#^p < 0.05, ^###^p < 0.0001; ^*^p < 0.05, ^**^p < 0.01, ^***^p < 0.0001, ANCOVA, N=6. “ns”, not significant.

As we expected, there is a significant decrease in endogenous IL-8 levels in the RelA-deficient line compared to the cells with RelA added back (Vector-DMSO vs C13-DMSO, p<0.01). IL-8 mRNA levels were significantly decreased in response to APX3330 and APX2009 in both Vector and C13 cells with respect to DMSO controls (p<0.05, **Figure 2A**). However, the effects were even more dramatic in Vector as IL-8 levels were minimally detectable following Ref-1 inhibition in RelA-deficient lines (p<0.001 vs Vector DMSO). Interestingly, IL-8 expression is not solely dependent on RelA as we observed some expression in the RelA-deficient cells (Vector DMSO), and yet blockade of Ref-1 signaling in these cells was able to almost completely abrogate the expression of IL-8 (Vector APX3330 and Vector APX2009), suggesting that the other TFs regulating its expression are also under Ref-1 redox control.

Surprisingly, the basal levels of both FOSB and c-Jun were much higher (~4-fold) in RelA-deficient lines (Vector DMSO vs C13 DMSO, p<0.05) (**Figures 2B, C**). Treatment with APX3330 and APX2009 resulted in strong upregulation of FOSB in RelA-deficient cells (Vector DMSO vs Vector APX, p<0.001), however in cells with functional RelA, Ref-1 inhibition largely abrogated this induction (Vector APX3330 vs C13 APX3330, p<0.001; Vector APX2009 vs C13 APX2009, p<0.001). Similar results were observed for c-Jun except that treatment with APX3330 could block the induction of c-Jun in the Vector control cells (Vector DMSO vs Vector APX3330, p<0.001).This data suggests that RelA signaling may be promoting transcription of a repressor or there is dysregulation of the AP-1 –RelA crosstalk resulting in loss of a negative feedback loop.

### STAT3 Is Not a Primary Target Determining Cellular Sensitivity to Ref-1 Inhibitors in Murine PDAC Kras^G12D^ Cells

PDAC pathways are significantly altered when Ref-1 expression is decreased including the STAT3 signaling pathway (14). We previously demonstrated the synergistic effects of dual targeting Ref-1/STAT3 axis in PDAC *in vivo* xenograft model and in KPC tumor cells (13). Therefore, we expanded our investigations to examine if other TFs that are regulated by Ref-1, such as STAT3 also contribute to the sensitivity of cells that are driven by Kras and yet do not express functional RelA. In these studies, STAT3 levels were reduced using siRNA in the KC3590 cell line series to evaluate the cellular sensitivity to Ref-1 inhibitors.

**Figure 3:**
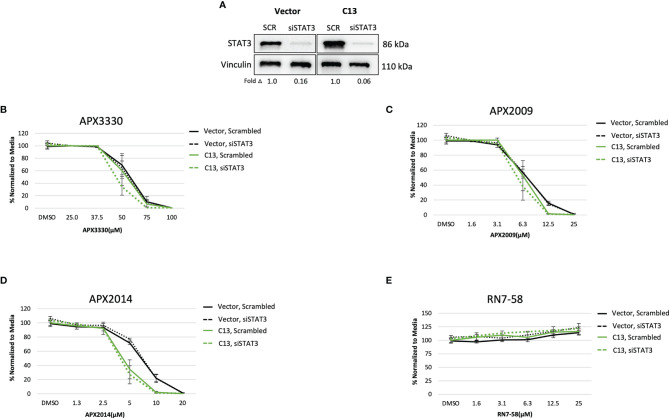
STAT3 is not a primary target altering cellular sensitivity to Ref-1 inhibitors in murine PDAC Kras^G12D^ cells. **(A)** STAT3 was knocked down in KC3590 cells that are RelA deficient (Vector) and clone with functional RelA add back (C13) and knockdown efficiency was assessed by Western bot. Vinculin was used as loading control. The cells were challenged for 48 hours with **(B)** APX3330, **(C)** APX2009, **(D)** APX2014, and **(E)** RN7-58. Cytotoxicity was measured by alamarBlue. At least three independent experiments were performed (N = 3).

Upon STAT3 knockdown in both Vector and C13 cells (**Figure 3A**), the cellular response was identical following treatment with APX3330 and APX2009, with slight enhancement of APX2014 in the RelA-proficient cells, despite STAT3 knockdown of greater than 90% (**Figures 3B**–**D**). Minimal cell killing was observed in any of the conditions when cells were challenged with inactive analogue RN7-58 (**Figure 3E**). These results suggest that STAT3 minimally contributes to the cellular sensitivity to Ref-1 redox inhibition in this PDAC cell model; i.e. functional RelA is driving the response to Ref-1 inhibitors.

### Targeting the PRDX1/Ref-1 Axis Enhanced Cellular Sensitivity to Ref-1 Inhibition

**Figure 4:**
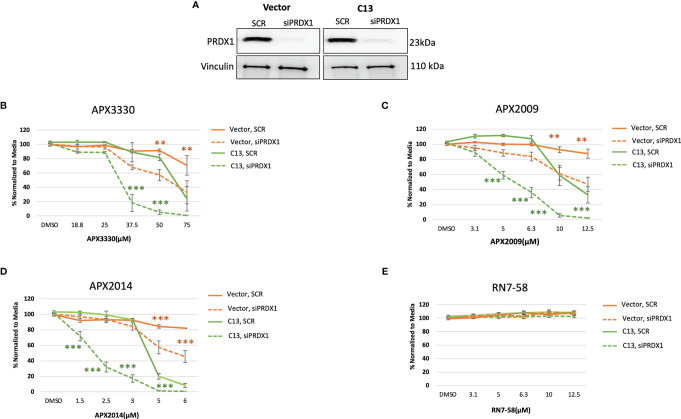
PRDX1 is an effective target enhancing cellular sensitivity to Ref-1 inhibitors in mouse PDAC cells. **(A)** PRDX1 knockdown efficiency in KC3590 Vector and C13 cell lines were greater than 80% comparing to scrambled control (SCR). Vinculin was used as loading control. **(B–D)** The cytotoxicity of Ref-1 inhibitors, APX3330, APX2009, APX2014 upon the condition of PRDX1 knockdown both in Vector and C13 were assessed. **(E)** cytotoxicity of RN7-58 was also evaluated along with Ref-1 inhibitors. Two-way ANOVA, **p < 0.01, ***p < 0.001. At least three independent experiments were performed (N = 3).

Previous studies demonstrated signaling interactions between PRDX1 and Ref-1 that led to changes in IL-8 levels, presumably through RelA (15). Thus, we wanted to determine if perturbation of PRDX1, an oxidizer of Ref-1, could alter cellular responses to Ref-1 redox inhibitors observed in the KC3590 cell. Again using siRNA, PRDX1 levels were reduced to greater than 80% in both Vector and C13 lines (**Figure 4A**). Upon PRDX1 knockdown, dramatic enhancement of cellular sensitivity to APX3330, APX2009, and APX2014, in both Vector and C13 cells was observed in comparison to scrambled controls (**Figures 4B**–**D**). Surprisingly, the enhancement in cellular sensitivity was nearly 2-fold more in RelA-proficient cells compared to the RelA-deficient cells, demonstrating RelA-dependent effects on cellular responses to Ref-1 redox signaling inhibition and imbalance of redox homeostasis through knockdown of PRDX1. We did not observe any differential cell killing when Vector and C13 cell lines were challenged with an inactive analog RN7-58, confirming inhibitor specificity (**Figure 4E**). These results clearly indicate that PRDX1 is a key mediator impacting cellular sensitivity to Ref-1 redox inhibition, and these cellular responses are much more effective in the presence of functional RelA.

### Targeting PRDX1/Ref-1 Redox Cycling With Ref-1 Inhibition and Its Impact on Cellular Redox Homeostasis

**Figure 5:**
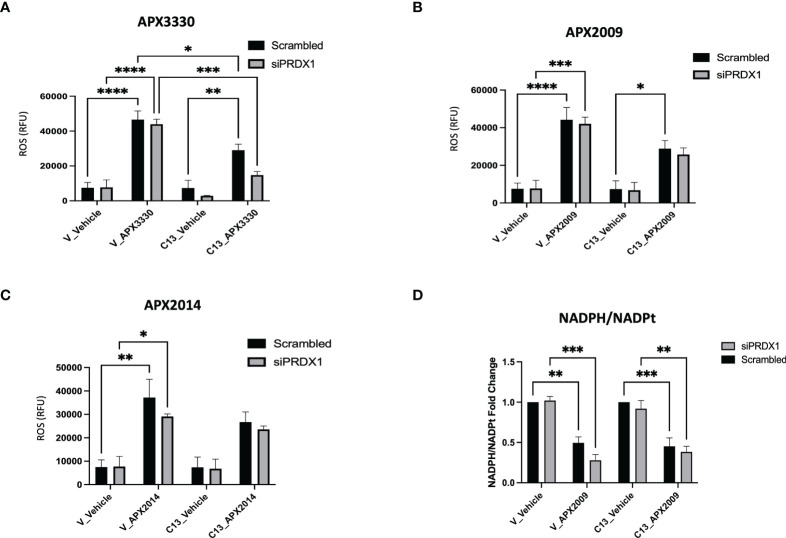
Effects of RelA on cellular redox imbalances caused by Ref-1 inhibitors in PDAC cells. **(A–C)** intracellular ROS levels were measured upon PRDX1 knockdown cells at 48 hours post-transfection and following 2 hours of Ref-1 inhibitor treatment with APX3330 (75 μM), APX2009 (20 μM), APX2014 (20 μM). These experiments were repeated four times (N=4), and then compared by Two-way ANOVA of *p < 0.05, **p < 0.01, ***P < 0.001, ****p < 0.0001. **(D)** NADPH/NADPt ratio in PRDX1 knockdown cells (1nM/48h) was assessed following APX2009 treatment (12.5µM/5h). two-way ANOVA, **p < 0.01, ***p < 0.001, N=3.

Two potential mechanisms that could explain the dramatic results on cell growth in **Figure 4** as well as relate to Ref-1/PRDX1/RelA signaling are generation of reactive oxygen species (ROS) and changes in redox status of the cell. To determine whether intracellular ROS production may be a part of the mechanism of RelA-driven differential cellular responses to Ref-1 inhibitors, we measured ROS levels, specifically superoxide and/or hydroxyl radicals, after PRDX1 knockdown and Ref-1 inhibitor treatment in KC3590 cells. Consistent with our previous findings, Ref-1 inhibition *via* APX3330, APX2009, or APX2014 generated significant amounts of ROS in the Vector lines, regardless of the levels of PRDX1 compared to vehicle controls (**Figures 5A, B**). Similar effects were observed with APX3330 and APX2009 in RelA-proficient cells, except following treatment with APX2014. There is a trend that there is increased ROS with APX2014 in the RelA-proficient cells, but it did not reach significance (**Figure 5C**). Importantly, the reduced expression of PRDX1 did not result in changes in ROS levels in untreated or treated Vector or C13 cells, which could be due to the species of ROS detected with the CellRox green assay. This data does show that superoxide and/or hydroxyl radical generation are not driving the large increase in cell killing seen with PRDX1 knockdown and Ref-1 inhibition in RelA-proficient cells.

To investigate the redox imbalance induced by Ref-1 inhibitors and PRDX1 knockdown and the link to RelA function in the cells, we assessed NADPH/NADP ratios following APX2009 treatment in cells with reduced expression of PRDX1. KC3590 cells treated with Ref-1 inhibitor, APX2009 display markedly reduced levels of NADPH as observed from the NADPH/NADP ratio in both Vector and C13 (**Figure 5D**). This indicates a more oxidized environment as expected after blocking Ref-1 redox function (25). Although Ref-1 inhibition resulted in a shift in the redox status of the cell, neither PRDX1 nor RelA expression altered this result. Again selective knockdown of PRDX1 is insufficient to change the generic redox balance in this matched cell line *in vitro* model pointing toward another mechanism of enhancement of cell killing in the KC3590 RelA-proficient cells.

## Discussion

Our studies described here investigate the RelA-driven cellular responses to Ref-1 redox inhibition through Ref-1/PRDX1 redox signaling in mouse PDAC cells. RelA has been implicated in driving resistance to treatments such as radiation and Gemcitabine. In one study, transiently silenced RelA increased Gemcitabine-induced cell killing (31), while in another study selective knockdown of RelA in combination with pyruvate dehydrogenase kinase (PDK1/2) enhanced radiation sensitivity of pancreatic cancer cells (32). RelA activity is regulated in many ways including redox regulation by Ref-1 (19, 33). Ref-1’s activation of transcription factors such as RelA can be influenced by PRDX1, a peroxidase in the Ref-1/TRX1 redox cycling pathway (15). Results presented here demonstrate our novel observation that pancreatic cancer cells become more sensitive to Ref-1 redox inhibition when PRDX1 expression is decreased and when RelA is present in the cells indicating a novel interplay between PRDX1, Ref-1, and RelA.

In these studies, we used a murine PDAC cell line KC3590 with Kras^G12D^ and a truncated RelA gene with missing exons 7-10 (KC) (**Supplementary Figure 2**). Exons 7-10 encode Rel homology domain (RHD), which is essential for dimerization of RelA, nuclear translocation, and DNA binding (22) (21). KC3590 cells were transfected with pcDNA3-Flag-RelA (clones C13 and C15) to have matched lines that express functional RelA and non-functional RelA. Due to Ref-1’s redox regulation of NF-κB, these lines were used as an important tool to interrogate the cells’ response to established Ref-1 inhibitor APX3330 as well as new analogs. APX3330, as well as the second-generation analogues APX2009 and APX2014 have been shown to be specific for Ref-1, directly targeting and interacting with the protein and not the downstream TFs (13, 34–39). This specificity has also been validated using another analogue of the APX compounds, RN7-58 which is similar in structure and came from the same structure-activity relationship (SAR) studies but is inactive in blocking Ref-1 redox activity (40, 41). As shown in **Figures 1A**–**C**, we demonstrated that RelA functionally deficient cells were more resistant to Ref-1 inhibitors APX3330, APX2009, and APX2014 and ranked in the top three Ref-1 inhibitor-resistant phenotype along with KC6075 (**Supplementary Table 2**). However, KC3590 cells with RelA added back were found to be significantly more sensitive to all three Ref-1 inhibitors. Accordingly, KC3590 C13 and C15 cells were in the top three Ref-1-inhibitor sensitive lines and ranked along with KC5748. These data support that our Ref-1 redox inhibitors are indeed hitting predicted downstream targets of Ref-1 i.e., NF-κB/RelA and that at least part of the mechanism of cell killing is mediated through RelA. Furthermore, we confirmed that the RelA-dependent differential cellular responses were lost when treated with a Ref-1 endonuclease specific inhibitor ARi3 (APE1/Ref-1 DNA Repair Inhibitor III). Additionally, an inactive analog of the APX redox inhibitor compounds, RN7-58 demonstrated no differential response or activity (**Figure 1D**). These findings once again underscore that the redox function of Ref-1 and subsequent regulation of RelA, but not the DNA repair function, plays a crucial role in driving cellular responses to Ref-1 redox inhibition in this particular cell model.

We also looked at another transcription factor that is under Ref-1 redox control, STAT3, to determine whether its expression correlates with the cytotoxicity response to Ref-1 redox inhibitors. Interestingly, we did not observe any differential cellular responses in cells with STAT3 knocked down when challenged with the Ref-1 redox inhibitors (**Figure 3**). In our previous studies, dual targeting of STAT3 with STAT3 inhibitors, Ruxolitinib or Napabucasin, along with Ref-1 redox inhibitors significantly increased cell killing in multiple PDAC cell lines (28). Additionally, we demonstrated that KPC cells (*LSL-Kras^G12D/+^;LSL-Trp53^R172H/+^;Pdx-1-Cre*) that lack expression of IL-6 and thereby reduced STAT3 signaling are very sensitive to the effects of Ref-1 redox inhibition. These cells have a mutated p53 while the murine PDAC cells used here do not. It is possible that p53 is important in the response to Ref-1 inhibition as p53 is also a redox target of Ref-1. These data also suggest a much more complex interplay between the genetic makeup of the tumors and the response to targeted agents – a focus of future studies. These differences support the well-established heterogeneity that exists in pancreatic cancer. Regardless, the results presented here support the rationale that RelA, but not STAT3, is a primary target in determining mouse PDAC cellular responses to Ref-1 redox inhibition in this Kras^G12D^ model.

Several *in vitro* studies reported that targeting redox activity of Ref-1 by APX3330 blocks activation of inflammatory modulators, such as RelA, IL-8 in human cancer lines (15, 19). We hypothesized that RelA deficient and proficient cells would demonstrate differential inflammatory responses to Ref-1 inhibitors. Indeed, IL-8 gene expression was significantly reduced in the RelA-deficient cells compared to proficient and in response to Ref-1 inhibitor treatments in both cell lines (**Figure 2A**). However, additional TFs must regulate IL-8 in these cells because IL-8 was still detectable in the deficient cells. IL-8 has been reported to be regulated by both NF-κB and AP-1 which could explain the lack of expression of IL-8 in the RelA-deficient cells following treatment with APX3330 and APX2009 (42). This data supports RelA driving IL-8 expression and that Ref-1 inhibition can block the activity of RelA and the other potential TFs that regulate IL-8 leading to very dramatic decreases in IL-8.

Additionally, we found 4-fold increased expression of FOSB and c-Jun mRNA levels in the Vector, RelA deficient cells compared to cells expressing RelA, C13 (**Figures 2B, C**), suggesting that RelA drives the expression of a repressor of FOSB and c-Jun or perturbation of some unknown feedback loop. The levels of FOSB were dramatically increased (20-40-fold) in response to Ref-1 redox inhibitors compared to Vector untreated control (**Figure 2B**). Studies have demonstrated that high expression AP-1 family proteins are involved in resistance to therapy to anti-cancer agents (43, 44). Higher expression of FOSB and c-Jun levels correlated to resistance to Ref-1 redox inhibitors as well. We also showed that in the presence of functional RelA, Ref-1 inhibitors at least in part restored FOSB and c-Jun mRNA expression to the control levels observed in C13 untreated cells (**Figures 2B, C**). These findings revealed that the added back functional RelA suppressed FOSB as well as c-Jun mRNA levels and may play a role in the sensitization of cells to Ref-1 redox inhibitors. As with the IL-8 data, AP-1 family members may also be playing a role in this response to Ref-1 inhibitors. AP-1 and NF-κB TFs can crosstalk and influence expression of various AP-1 family members (30).

Additionally, we learned that PRDX1 is playing an important role in the cellular response to Ref-1 inhibitors. Upon knocking down PRDX1, KC3590 cells were much more sensitive to Ref-1 redox inhibitors. Interestingly, the effects were significantly stronger when functional RelA was present in the cells (**Figure 4**). To dissect the role of RelA in Ref-1/PRDX-1 redox signaling, we examined ROS levels in response to Ref-1 inhibition. Indeed, we observed higher levels of ROS with Ref-1 inhibitor treatments, as we previously reported in human PDAC cells (28). Although PRDX1 levels did not influence ROS levels in either RelA-deficient or -proficient lines, RelA-deficient lines tended to have higher amounts of ROS in response to Ref-1 inhibitors when comparing to RelA-proficient lines. This difference was more prominent with APX3330, a quinone-based structure, compared to APX2009 and APX2014 which are naphthoquinone (**Figures 5A–C**). One caveat to this study is that the ROS assay that was utilized will not detect all forms of ROS so there is the possibility that PRDX1/Ref-1/RelA axis is altering a different ROS species that was not detected in the CellRox green assay. Moreover, we found a significant reduction in NADPH/NADP ratios with APX2009 in both RelA-proficient and -deficient cell lines indicative of a shift in redox balance toward a more oxidized state. Surprisingly, reducing expression of PRDX1 was insufficient to alter the generic redox balance (**Figure 5D**). Future work will delineate more specific details of redox mechanism of Ref-1/PRDX1/2 axis in the cellular response to Ref-1 inhibition. These studies will further delineate the relationship of cellular redox cycling pathways and their role in regulating Ref-1 redox activity as well as potential translational significance. APX3330 has been in over 300 patients in clinical trials spanning diseases from hepatitis to oncology and currently enrolled in a phase II trial in diabetic retinopathy and diabetic macular edema. In all of the trials to date, it has demonstrated a strong safety profile.

Although many studies have shown a role for NF-kB/RelA in PDAC inflammatory responses and therapy resistance, little is known as to how these inflammatory responses are modulated through redox signaling pathways in PDAC and its impact on sensitivity to PDAC treatment regimens. While the role of Ref-1 and NF-κB and Ref-1’s redox signaling inhibition has been supported in other inflammatory model systems, as observed in the conversion of preleukemia to leukemia (45), inflammatory bowel disease (46), and retinal indications (47), the uniqueness of these studies is the use of a genetically modified murine KC cell model with functional mutation of NF-kB/RelA. This system was used to directly investigate RelA-mediated differential sensitivity to Ref-1 redox inhibitors and the regulation of inflammatory cytokines in response to Ref-1 redox signaling inhibition. We also uncovered dramatic enhancement in cell killing in response to Ref-1 redox inhibitors when PRDX1/Ref-1 redox cycling was blocked, especially in presence of RelA. This study has provided an insight into interactions between PRDX1/Ref-1 redox signaling and its inhibition by specific APX drugs which will further advance our push for new therapeutic strategies and improve anticancer drug efficacy in PDAC.

## Data Availability Statement

The raw data supporting the conclusions of this article will be made available by the authors, without undue reservation.

## Author Contributions

MM performed experiments and led writing of the manuscript. RW performed experiments and assays, as well as contributed figures and to the writing and editing. LA performed some cellular cytotoxicity studies. SG performed assays and writing. ZH provided us with numerous cell lines, expertise, and experiments. CS performed analysis as well as cells and editing. GS provided us with cells and expertise. CZ provided bioinformatic analysis. MF provided expertise, experimental design, and writing/editing. MK provided expertise, experimental design, analysis and writing/editing of manuscript. All authors contributed to the article and approved the submitted version.

## Funding

MK and MF were supported by grants from the National Institute of Health and National Cancer Institute R01CA167291 and R01CA254110. MK was also supported by NIH/NCI grants R01CA205166, R01CA231267, R01EY031939 and R01HL140961. MF was also supported by NIH/NCI grant U01HL143403, R01CA211098 and R01NF180045. MF and MK were additionally supported by the Riley Children’s Foundation and the IU Simon Comprehensive Cancer Center, P30CA082709.

## Conflict of Interest

MK has licensed APX3330 through Indiana University Research and Technology Corporation to Apexian Pharmaceuticals LLC.

The remaining authors declare that the research was conducted in the absence of any commercial or financial relationships that could be construed as a potential conflict of interest.

## Publisher’s Note

All claims expressed in this article are solely those of the authors and do not necessarily represent those of their affiliated organizations, or those of the publisher, the editors and the reviewers. Any product that may be evaluated in this article, or claim that may be made by its manufacturer, is not guaranteed or endorsed by the publisher.
