# Supplementary material for: RelA Is an Essential Target for Enhancing Cellular Responses to the DNA Repair/Ref-1 Redox Signaling Protein and Restoring Perturbated Cellular Redox Homeostasis in Mouse PDAC Cells
Source: Front Oncol. 2022 Mar 24;12:826617. doi: 10.3389/fonc.2022.826617 (PMC8988139; doi:10.3389/fonc.2022.826617)
Supplement: Supplementary file 1 [file DataSheet_1.docx]

Supplementary Material

**Table S1.** Primers list for quantitative RT- PCR analysis

| **Primers** | **Forward** | **Reverse** |
| --- | --- | --- |
| IL-8 | GGTGATATTCGAGACCATTTACTG | GCCAACAGTAGCCTTCACCCAT |
| FOSB | ACCTGTCTTCGGTGGACTCCTT | TGGCTGGTTGTGATTGCGGTGA |
| c-Jun | CAGTCCAGCAATGGGCACATCA | GGAAGCGTGTTCTGGCTATGCA |
| RPL6 | AGAAGGCTACGCTCCAGCATCA | CAGTCACAAGCAGCAAGCCACT |

| **Table S2**. Mouse PDAC cells (KC) are ranked based on EC_50_ (μM) from most to least resistant to Ref-1 inhibitors, APX3330, APX2009, and APX2014. | | | | |
| --- | --- | --- | --- | --- |
|  | **KC lines** | **APX3330** | **APX2009** | **APX2014** |
|  | KC6075 | 65.1 | 27.8 | 6.6 |
|  | KC8442 | 40.7 | 28.5 | 6.2 |
|  | **KC3590_P** | **60.8** | **16.8** | **6.0** |
|  | **KC3590_V** | **52.9** | **13.7** | **6.0** |
|  | KC2259 | 56.0 | 10.8 | 5.5 |
|  | KC53631 | 59.9 | 9.9 | 4.6 |
|  | KC9091 | 49.4 | 7.7 | 3.8 |
|  | KC5671 | 49.0 | 7.3 | 3.7 |
|  | KC5559 | 37.5 | 8.0 | 3.0 |
|  | **KC3590_C13** | **36.2** | **6.6** | **3.0** |
|  | **KC3590_C15** | **39.3** | **6.6** | **2.8** |
|  | KC5748 | 32.1 | 4.2 | 3.3 |
| Abbreviation of KC3590: Parent (P), Vector (V), Clone13 (C13), Clone15 (C15). | | | | |

***

***

***

**APX3330**

***

***

**

**APX2009**

***

*

**APX2014**

**Supplementary Figure 1.** Cytotoxicity of Ref-1 inhibitors resistant (KC6075) and sensitive (KC5748) cell line. KC cells were challenged with (A) APX3330, (B) APX2009, or (C) APX2014 in 5% serum media for 48 hours. Cytotoxicity was measured by alamarBlue. Student T- test, ^**^p<0.001, ^***^p<0.0001, At least five independent experiments were performed (N=5).

Parent

Vector

C13

KC9091

Full length RelA

(added back)

Delta RelA

(truncated)

110kDa

Vinculin

RelA


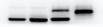

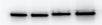

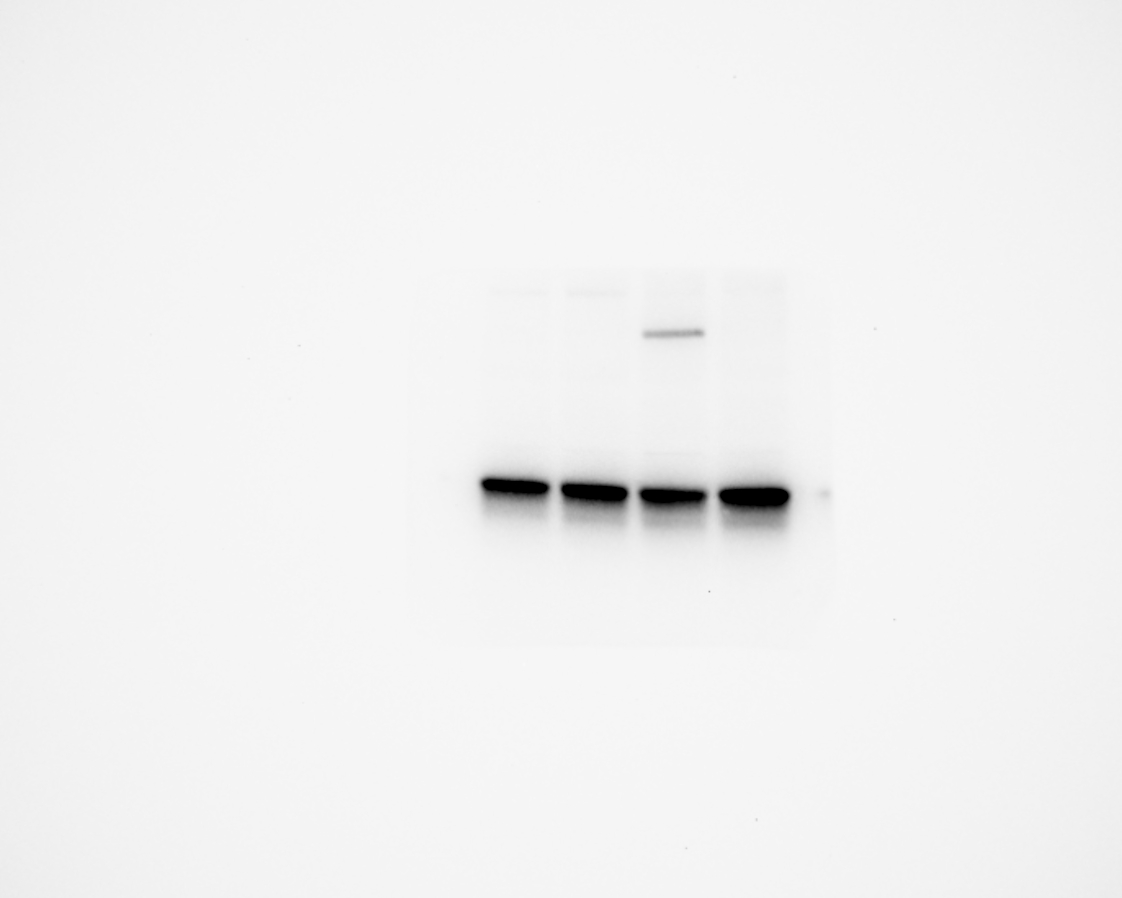


Ref-1

36kDa

KC3590

**Supplementary Figure 2.** Expression levels of RelA and Ref-1 in mouse PDAC cells.

The protein levels of RelA and Ref-1 in KC3590 cells and KC9091 lines were assessed by western blot. KC9091 lines express wild type (WT) RelA. Vinculin was used as loading control (N=3).

**Supplementary Figure 3.** FOSB and c-Jun gene expression profile in human PDAC (PaO3C) cells. RNA sequence data revealed the FOSB and c-Jun expression in PaO3C cells upon Ref-1 knockdown (siRef-1) conditions. Indices on the x-axis represent the single cells under the Ref-1 siRNA (red) and Control (blue) conditions and y-axis is the log (CPM+1) scaled gene expression level of c-Jun and FOSB.


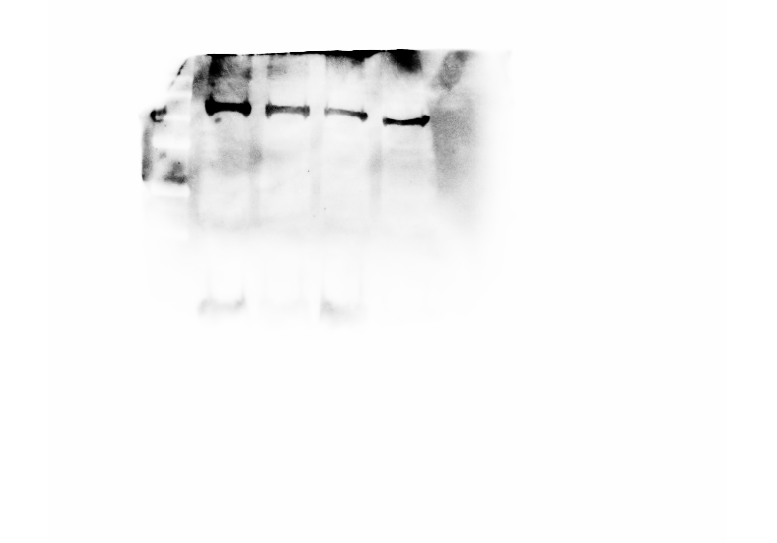

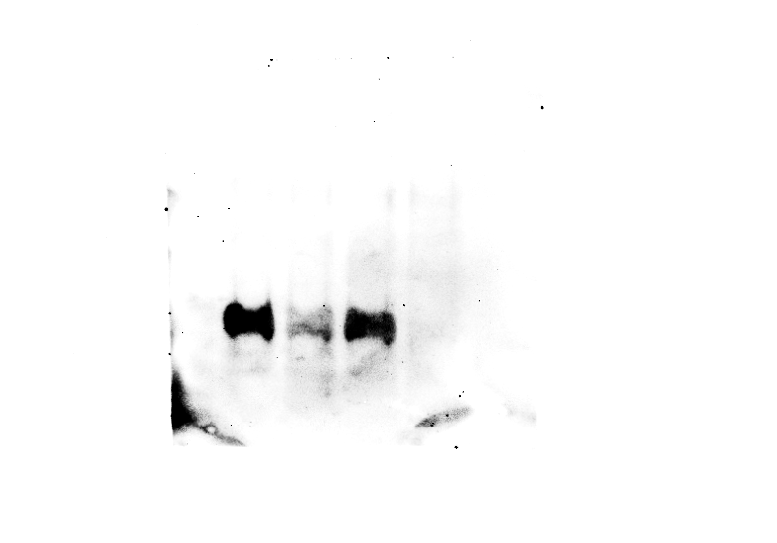


**Vector**

23 kDa

SCR

PRDX1

Vinculin

110 kDa

siPRDX1

SCR

siPRDX1

**C13**


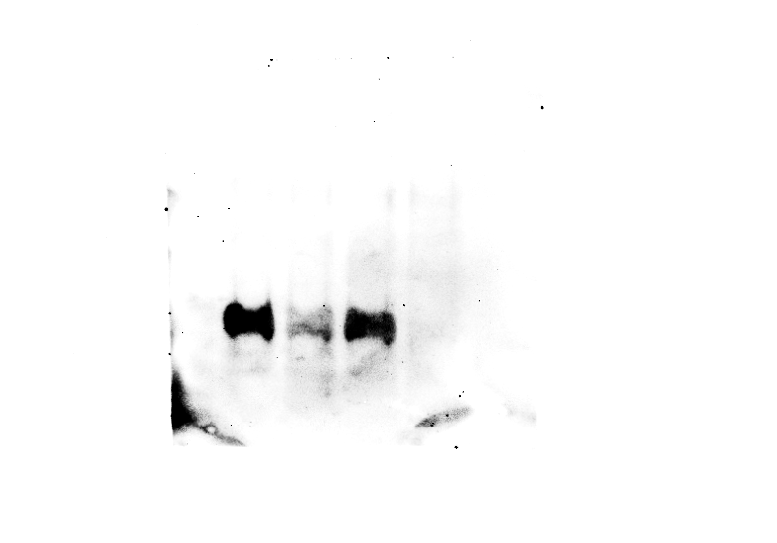

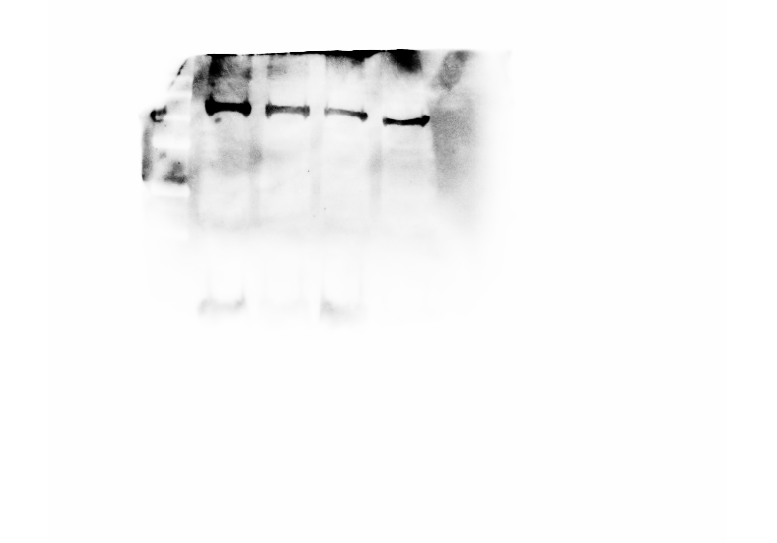


**Supplementary Figure 4.** PRDX1 protein expression in KC3590 Vector and C13 cells.

PRDX1 knock down in KC3590 Vector and C13 cells assessed by Western bot and knockdown efficiency was greater than 80% comparing to scrambled control (SCR). Vinculin was used as loading control (N=4).
